# COGNITIVE MISPERCEPTION, DISABILITY, AND MORTALITY AMONG OLDER ADULTS IN 25 COUNTRIES

**DOI:** 10.1093/geroni/igad104.0694

**Published:** 2023-12-21

**Authors:** Zhuoer Lin, Mark Schlesinger, Xi Chen

**Affiliations:** Yale University, New Haven, Connecticut, United States; Yale University, New Haven, Connecticut, United States; Yale University, New Haven, Connecticut, United States

## Abstract

Despite a large body of literature on cognitive ability and health, less is known about the health consequences of biased cognitive perception. Using harmonized and nationally representative longitudinal surveys from 25 countries spanning Asia, Europe and the Americas, we document the growing gap between actual and perceived cognitive ability that appears to increase with age, and construct a standardized measure of cognitive misperception. Linking this novel measure with mortality and disability, we model the cognitive misperception – health gradient. Results show that being in the upper quartile of cognitive misperception (i.e., showing heightened overconfidence in cognition) leads to higher mortality rates within 1 year, 3 years, and 5 years. Conditional on survival, being overconfident in cognition also greatly increases the risks of incident disability and frailty, especially for older adults receiving less family support. We identify two possible pathways inducing poorer outcomes: the first stemming from increased risk taking and financial vulnerability; the second associated with suboptimal use of preventive services and declines in health-promoting behaviors. Given the large and profound impacts of cognitive misperception on older adults’ health and well-being, more family and social supports, public investment in education and health literacy, and better healthcare access and affordability are needed to increase the timely awareness of cognitive decline.

